# A review of the inclusion of equity stratifiers for the measurement of health inequalities within health and social care data collections in Ireland

**DOI:** 10.1186/s12889-021-11717-5

**Published:** 2021-09-19

**Authors:** Christopher Carroll, Katie Evans, Khalifa Elmusharaf, Patrick O’Donnell, Anne Dee, Diarmuid O’Donovan, Marie Casey

**Affiliations:** 1 Department of Public Health HSE Mid-West, Mount Kennett House, Henry Street, Limerick City, Ireland; 2 Public Health Programme, School of Medicine, Faculty of Education and Health Services, Garraun, Castletroy, Co. Limerick, Limerick City, Ireland; 3 Centre for Public Health, Queen’s University Belfast, Royal Victoria Hospital, Grosvenor Road, Belfast, BT12 6BJ Northern Ireland

**Keywords:** Equity stratifiers, Dimensions of inequality, Health inequalities, Ireland

## Abstract

**Background:**

Health equity differs from the concept of health inequality by taking into consideration the fairness of an inequality. Inequities may be culturally specific, based on social relations within a society. Measuring these inequities often requires grouping individuals. These groupings can be termed equity stratifiers. The most common groupings affected by health inequalities are summarised by the acronym PROGRESS (Place of residence, Race, Occupation, Gender, Religion, Education, Socioeconomic status, Social capital). The aim of this review was to examine the use of equity stratifiers in routinely collected health and social care data collections in Ireland.

**Methods:**

One hundred and twenty data collections were identified from the Health Information and Quality Authority (HIQA) document, “Catalogue of national health and social care data collections: Version 3.0”. Managers of all the data collections included were contacted and a data dictionary was requested where one was not available via the HIQA website. Each of the data dictionaries available was reviewed to identify the equity stratifiers recorded.

**Results:**

Eighty-three of the 120 data collections were considered eligible to be included for review. Twenty-nine data dictionaries were made available. There was neither a data dictionary available nor a response to our query from data collection managers for twenty-three (27.7%) of the data collections eligible for inclusion. Data dictionaries were from national data collections, regional data collections and national surveys. All data dictionaries contained at least one of the PROGRESS equity stratifiers. National surveys included more equity stratifiers compared with national and regional data collections. Definitions used for recording social groups for the stratifiers examined lacked consistency.

**Conclusions:**

While there has been much discussion on tackling health inequalities in Ireland in recent years, health and social care data collections do not always record the social groupings that are most commonly affected. In order to address this, it is necessary to consider which equity stratifiers should be used for the Irish population and, subsequently, for agreed stratifiers to be incorporated into routine health data collection. These are lessons that can be shared internationally as other countries begin to address deficits in their use of equity stratifiers.

## Background

“Not everything that can be counted counts; not everything that counts can be counted” (attributed to Albert Einstein.)

A key element of improving population health is in understanding and measuring the causes of ill health in a society. However, we may be unaware of important causes of poor health if we do not or cannot measure them. According to Lynch, definitions of health inequalities have varied over time and have depended on their social context, but in general health inequalities refer to differences in health outcomes between population groups [1]. The ability to understand health inequalities depends on whether we recognise and measure them. At a population level, health indicators such as life expectancy may be incomplete if they do not account for the differences between population groups within a society.

### What are Equity Stratifiers?

Health equity differs from the concept of health inequality by introducing a judgement as to the fairness of an inequality. In her influential 1991 paper “The concepts and principles of equity in health”, Whitehead defined health inequities as differences in health that are unnecessary, avoidable, unfair and unjust [2]. Braveman and Gruskin argued for an idea of health equity that promotes social justice. They suggested that avoidability should not be considered a requirement in evaluating the presence of an inequity. They developed a working definition of equity in health for the purpose of measurement and study as:

*“The absence of systematic disparities in health (or in the major social determinants of health) between social groups who have different levels of social advantage/disadvantage – that is, different positions in a social hierarchy.”* [3] p254.

Equity stratifiers, also known as “dimensions of inequality”, are variables chosen to reflect a perceived inequality. They are often categorical in nature in order to detect structural inequalities by comparing aggregate data on groups of people. PROGRESS-PLUS summarises the equity stratifiers most frequently used in health inequality monitoring and they can be used as a tool to guide equity analyses [4].

PROGRESS stands for:

- Place of residence
- Race (or ethnicity)
- Occupation
- Gender (or sex)
- Religion
- Education
- Socioeconomic status
- Social capital

PROGRESS was initially developed as a tool for an analysis of victims of road traffic accidents [5]. The PLUS suffix was added to include personal characteristics, relationship features and time-specific stratifiers [6]. As a framework, PROGRESS-PLUS is endorsed and incorporated into the work of the Campbell and Cochrane Collaborations and PRISMA as a means to report on or analyse the impact of an intervention or practice on inequalities [4, 7].

At a global level, the World Health Assembly Resolution 62.14 “urges” member states “to monitor and measure the health of national populations, with disaggregated data such as age, gender, ethnicity, race, caste, occupation, education, income and employment where national law and context permits so that health inequities can be detected and the impact of policies on health equity measured” [8] p3. The language of equity stratifiers has also been adopted by the World Health Organisation (WHO) who have produced a step-by-step guide on the process of measuring health inequalities, which they describe as “an essential public health function” [9] p3. Their first steps include deciding on the health topics of interest, identifying health indicators to measure and choosing the corresponding equity stratifiers.

Neither PROGRESS-PLUS, nor the recommendations of the World Health Assembly should be considered a comprehensive list of equity stratifiers. The appropriate stratifiers to use will vary with social and cultural context. It is therefore necessary to develop a framework for choosing stratifiers which are culturally specific.

Some countries are more proactive than others in their commitment to measure health inequalities. In recent years the Canadian Institute of Health Information has produced two reports and a toolkit focused on defining equity stratifiers for a Canadian context [10, 11]. Their toolkit provides a guide for integrating equity stratifiers into the analysis and reporting of health inequalities.

Public Health England has developed a “Public Health Outcomes Framework” which comprises of a list of health-related indictors tracked over time to monitor their performance [12]. Eighteen indicators are specifically included to reflect the “Social Determinants of Health”. Each of these indicators are broken down by the equity stratifiers deemed appropriate and these can be tracked over time to examine trends such as widening inequalities. In 2020, the “Marmot Review 10 Years On” relied on this data to report on health inequalities in England and how they have changed since the initial Marmot Review was published [13].

Within an organisation the routine collection of data that incorporates equity stratifiers can also be used to examine the impact of the organisation’s structures or processes on outcomes pertaining to equity. For example, following on from a Health Equity Audit of their immunisation services using routinely collected data, Public Health England have recently published an “Immunisation Inequalities Strategy” [14]. This provides a systematic framework for identifying health inequalities within their service, developing an evidence based approach to address them and to monitoring progress going forward.

### Health Inequalities in Ireland

Lower socio-economic status is frequently associated with worse health outcomes in countries around the world. This was a central theme of the WHO’s “Closing the Gap in a Generation” report produced in 2008 by the Commission on Social Determinants of Health [15]. Health inequalities related to socio-economic status are also seen in the Irish context. In 2019, the Central Statistics Office (CSO) reported life expectancies broken down by socioeconomic status using an area level measure, the Pobal HP Deprivation Index. Based on data from persons who died in a twelve-month period between 2016 and 2017, they found males living in areas within the most deprived quintile of Ireland lived five years less than males in the most affluent quintile. Females lived 2.3 years less [16].

Examples of other inequalities reported using equity stratifiers in Ireland include disability, language, homelessness and ethnicity. Based on CSO data published in 2019, people living with a disability had a standardised death rate more than four times that of those without a disability [16]. Migrants have been found to have inadequate access to interpreters/translators for health care consultations [17]. People who are homeless and rough sleeping have an increased mortality rate. A study from Dublin found rough sleepers had an average age of death of only 42 years for men and 38 years for women [18]. Irish Travellers have worse health outcomes compared with the settled population. In the All Ireland Traveller Health Study published in 2010 male life expectancy was 15.1 years lower than the general population and female life expectancy was 11.5 years lower [19].

However, these examples belie the use of routinely collected data for measuring health inequalities. Much of our evidence demonstrating health inequalities comes from once off research which requires funding and time, meaning significant inequalities are only reported on years after the data was available. There is considerably more information on health inequalities that we could collect and use if we included more equity stratifiers consistently in our routine health data collection. The WHO recommends that health inequalities are considered in routine data collection as the first step to addressing them [9]. Thereafter, it is important that we have an evaluation framework that can monitor trends in inequalities following policy changes or interventions.

In Section 42 of the Irish Human Rights and Equality Commission Act 2014, known as the Public Sector Duty, there is a requirement for public bodies to promote equality, prevent discrimination, and protect the human rights of its members, staff and the persons to whom it provides services [20]. This requires consensus among policy makers, data owners and practitioners, on standard measures. From the perspective of the Health Service Executive (HSE), the national body that provides publicly funded health and social care services, this duty has important implications for addressing health inequalities, and will need strategic planning and the consideration of the equity stratifiers that will be used to measure and promote equality.

The aim of this study was to explore the use of the equity stratifiers in routinely collected Irish health and social care data collections.

## Methods

### Study Design

One hundred and twenty data collections were identified from the Health Information and Quality Authority (HIQA) document; “Catalogue of national health and social care data collections: Version 3.0” [21]. In Ireland, HIQA is the statutory authority charged with monitoring the collection of health and social care data. Specifically, under the Health Act 2007, HIQA has a legislative responsibility to set standards for the HSE with regards the data and information it collects and to advise the Minister for Health as to compliance with these standards [22].

HIQA’s catalogue groups data collections into six categories: national data collections, regional data collections, national censuses, national surveys, national performance reports and additional sources of health information. Collated sources of health and social care information and data collections which do not record individual level health data were not considered for inclusion in the analysis.

### Data Collection

For each collection, the HIQA catalogue includes information on the availability of a data dictionary and contact details for the managing organisation. In some cases direct download links to data dictionaries are provided. Initially, each collection was screened for a data dictionary and 13 were identified. Between February and July 2018 each of the managers, or equivalent, of the respective data collections was contacted by phone or email to enquire whether they had a data dictionary that they were willing to share. Where no reply was received from the manager of a data collection following email correspondence, a follow up phone call was made to ensure the email address was correct and to further explain the purpose of the request.

All data managers were also asked if they had considered equity stratifiers and their comments were noted.

Two of the authors (CC, KEv) independently decided on the presence or absence of the specific PROGRESS stratifiers for each of the data dictionaries reviewed. Where disagreement occurred this was resolved by consensus with a third author (MC).

### Defining Stratifiers in the Irish Context

The presence or absence of stratifiers within a data dictionary was recorded overall. Additional levels of detail were included as appropriate. This included several specific terms for the recording of stratifiers which were unique to an Irish context.

For example, the most precise level of measurement of place of residence was an individual’s postcode, termed their “Eircode”. The Irish Central Statistics Office (CSO) datasets cover national areas which can be disaggregated into regional areas, county levels, electoral districts or small areas. There were also specific geographical areas which related to hospital or community healthcare service boundaries which are not commented on further.

Irish Travellers are an indigenous ethnic population in Ireland. It has been recognised that Travellers have worse health outcomes compared with the settled population and therefore they are an important ethnic group when considering inequalities [19].

The Pobal HP Deprivation Index developed by Trutz Haase and Jonathan Pratschke is a composite area based measure of socio-economic status which uses data from the national census. It has been internally validated and is commonly used in Ireland as a measure of socio-economic status [23].

### Research Ethics

No personal data from data collections were collected as part of this research. All managers were approached with the understanding that we were undertaking a research project and that their comments would contribute to its results. Comments collected from managers of individual data collections were anonymised. All correspondence was processed in accordance with HSE data protection policy [24]. Research conducted adhered to relevant Irish data protection legislation including the Health Research Regulations 2018 and the General Data Protection Regulation [25, 26].

## Results

### Data Dictionaries Included

**Fig. 1:**
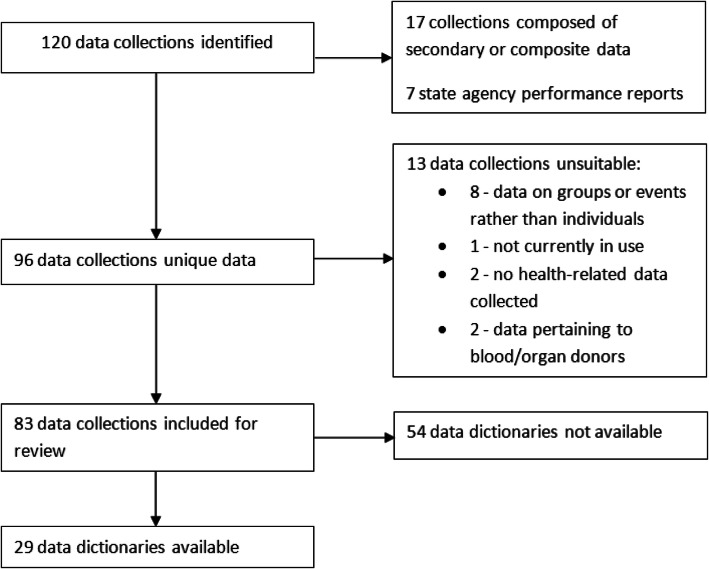
Flow diagram of the process for inclusion of data dictionaries

Of the 120 data collections in HIQA’s catalogue, 83 were considered eligible for inclusion. Figure 1 contains a flow diagram that summarises the reasons for exclusion of some data collections. Those reasons included collections not recording individual level data, collections no longer in use, collections using composite data from another source and collections with individual level data but which either has no health-related information or information that pertains to a donor rather than a patient.

Of the 83 data collections identified for inclusion, 29 (34.9%) data dictionaries were available for review. Nineteen were from national data collections, four were from regional data collections and six were from national surveys. Of the 54 data dictionaries which were not available, 23 data collections did not fully respond to our query, 19 data collections stated they did not have a data dictionary to share, nine data collections responded stating that their data dictionaries were under review and three data collections declined to send us their data dictionaries. This resulted in a non-response rate to our query of 27.7% of all collections eligible for inclusion, 23 of 83 data collections.

### Number of Stratifiers Identified

A breakdown of the proportion of data dictionaries which recorded each of the main PROGRESS-PLUS equity stratifiers can be found in Table 1. The equity stratifiers which were least frequently recorded were sexual orientation and religion. All data dictionaries contained measures of gender and age. Twenty-one (72.4%) data collections recorded place of residence. A measure of socio-economic status was identified in 16 (55.2%) data dictionaries overall, but in only seven (36.8%) of the national data collections. Race or ethnicity was recorded in 13 (44.8%) data dictionaries. Table 2 contains a list of the data collections for which a data dictionary was available and the equity stratifiers identified.

**Table 1:** Breakdown of percentage of data collections recording equity stratifiers by type of collection

Equity Stratifier	All (***n*** = 29)		National Collections (***n*** = 19)		Regional Collections (***n*** = 4)		National Surveys (***n*** = 6)	
	n	%	n	%	n	%	n	%
**Place of residence**	21	72.4%	11	57.9%	4	100.0%	6	100.0%
**Race/Ethnicity**	13	44.8%	8	42.1%	0	0.0%	5	83.3%
**Occupation**	15	51.7%	5	26.3%	4	100.0%	6	100.0%
**Gender**	29	100.0%	19	100.0%	4	100.0%	6	100.0%
**Religion**	3	10.3%	0	0.0%	0	0.0%	3	50.0%
**Education**	11	37.9%	3	15.8%	3	75.0%	5	83.3%
**Socio-Economic Status**	16	55.2%	7	36.8%	3	75.0%	6	100.0%
**Social Capital**	12	41.4%	6	31.6%	0	0.0%	6	100.0%
**Age**	29	100.0%	19	100.0%	4	100.0%	6	100.0%
**Disability**	9	31.0%	3	15.8%	0	0.0%	6	100.0%
**Sexual Orientation**	2	6.9%	2	10.5%	0	0.0%	0	0.0%

**Table 2:** PROGRESS equity stratifiers by data dictionary

	Place of Residence	Race/ Ethnicity	Occupation	Gender	Religion	Education	Socioeconomic status	Social Capital	Age	Disability	Sexual Orientation
**National Data Collections**											
Administration of Electro-convulsive Therapy in Approved Centres				✓					✓		
Admissions of Children to Approved Centres				✓					✓		
Cystic Fibrosis Registry of Ireland	✓	✓		✓		✓		✓	✓	✓	
Deaths relating to all residents in Approved Centres				✓					✓		
Hospital In-Patient Enquiry	✓			✓				✓	✓		
Irish National Rare Kidney Disease Registry		✓	✓	✓			✓		✓		
National Cancer Registry Ireland	✓		✓	✓			✓		✓		
National Drug Treatment Reporting System	✓	✓		✓		✓	✓	✓	✓		✓
National Drug-Related Deaths Index	✓	✓	✓	✓			✓		✓		✓
National Intellectual Disability Database	✓			✓					✓	✓	
National Paediatric Mortality Register		✓		✓		✓	✓	✓	✓		
National Perinatal Reporting System	✓	✓	✓	✓			✓	✓	✓		
National Physical and Sensory Disability Database	✓			✓					✓	✓	
National Poisons Information Centre Database				✓					✓		
National Psychiatric Inpatient Reporting System	✓	✓	✓	✓			✓	✓	✓		
National Registry of Deliberate Self Harm Ireland	✓			✓					✓		
Out of Hospital Cardiac Arrest Register	✓			✓					✓		
Use of Seclusion, Mechanical Restraint and Physical Restraint in Approved Centres				✓					✓		
Very Low Birth Weight Infants in the Republic of Ireland		✓		✓					✓		
**Regional Data Collections**											
EUROCAT European Registries of Congenital Anomalies	✓		✓	✓		✓	✓		✓		
EUROCAT European Registries of Congenital Anomalies	✓		✓	✓		✓	✓		✓		
EUROCAT European Registries of Congenital Anomalies	✓		✓	✓		✓	✓		✓		
Heartwatch	✓		✓	✓					✓		
**National Surveys**											
European Social Survey	✓	✓	✓	✓	✓	✓	✓	✓	✓	✓	
Growing Up in Ireland	✓	✓	✓	✓	✓	✓	✓	✓	✓	✓	
Health Behaviour in School Aged Children	✓	✓	✓	✓			✓	✓	✓	✓	
Healthy Ireland	✓	✓	✓	✓		✓	✓	✓	✓	✓	
Lifeways Cross-Generation Cohort Study	✓		✓	✓		✓	✓	✓	✓	✓	
The Irish Longitudinal Study on Ageing	✓	✓	✓	✓	✓	✓	✓	✓	✓	✓	

All data dictionaries contained at least one PROGRESS equity stratifier. National and regional data collections contained less equity stratifiers compared with national surveys. The median number of PROGRESS stratifiers contained within national data collections was 3 (range 1–6), in regional data collections was 5 (range 3–5) and in national surveys was 7.5 (range 6–8).

### PROGRESS Stratifiers

#### Place of Residence

A measure that related to place of residence was coded for in 18 of the 29 data dictionaries reviewed. Only five data collections recorded area of residence at a District Electoral Division (DED) level or smaller. Living in an urban or rural setting was recorded in the five data dictionaries, all from national surveys. All of the national surveys included a variable relating to place of residence, however only two recorded a specific location such as a county, DED, Small Area or Eircode.

#### Race/Ethnicity

A variable describing race or ethnicity was for coded for in 13 of the data dictionaries reviewed. Irish Traveller ethnicity was specifically recorded in nine data dictionaries. The coding options used were heterogeneous. Most used a combination of the terms used in the census by the CSO including Irish, white, black, Asian, African and Traveller. However, the European Social Survey did not use terms such as white or black, rather it records a specific ethnic group that a person belongs to, which is in most cases a nationality. The European Social Survey also uniquely records two levels of ethnicity for those with two ethnic backgrounds.

In addition to or instead of coding for race or ethnicity directly, a person or their guardian’s country of birth was recorded in eleven of the 29 data dictionaries. In correspondence from one national data collection manager it was stated that they had previously collected nationality but had been requested instead to record language and country of birth for service planning reasons.

#### Occupation

Fifteen of the 29 data dictionaries reviewed recorded occupation. Four out of the five national data collections which recorded occupation used the CSO classification. All six of the national surveys recorded occupation. Two incorporated the International Standard Classification of Occupations (ISCO). The European Social Survey included questions on four different aspects of occupation which, along with incorporating the ISCO classification, considered measures of class position from both a Weberian and a Marxian perspective [27]. Employment status was recorded in 14 of the 29 data dictionaries reviewed.

#### Gender

All data dictionaries reviewed contained a variable recording sex or gender. Fifteen of the data collections used a binary option of male or female. Four collections included “indeterminate” as an option. They were all perinatal data collections. Two data collections collected a variable for non-cisgender persons. Both these collections, managed by the same organisation, began recording non cisgender options and sexual orientation in recent years following requests from representative LGBTQ+ (lesbian, gay, bisexual, transgender, queer and others) groups.

#### Religion

Religion was recorded in three of the survey data dictionaries reviewed. Each used a different method of coding. They varied by the number of religions offered as options and whether no religion was a recorded answer.

#### Education

Education was recorded as a variable in eleven of 29 data dictionaries reviewed. The degree of detail varied from routine national data collections and national surveys. For the most part, the level of education recorded in the national data collections was based on the highest level completed going from primary to secondary to higher level. The type of higher level education was also often recorded. Parental education, either of the mother or of both parents, was recorded in data collections relating to children. Two data dictionaries recorded the age at which the respondent left education, along with the level reached.

Most of the national surveys recorded the educational attainment of more than one individual in the household or extended family. They generally included more detail as to the exact type of education received. The national survey with the most detailed collection of educational attainment gave 14 coding options.

#### Socio-Economic Status

Sixteen data dictionaries collected a measure of socioeconomic status. Ten of the 23 national and regional data collections contained a measure of SES. One was based on education alone, seven were based on occupation alone, one was based on place of residence alone and one was a composite measure of education and employment status. The area based measure of socio-economic status used the Pobal HP Deprivation Index [23].

Socioeconomic status is frequently derived from a combination of more basic variables. One data collection, the National Paediatric Mortality Registrar, had developed its own score of social deprivation relevant to their population. Variables included household employment, medical card status, a mother in receipt of social welfare, car ownership and home owner status.

Socioeconomic status was sometimes calculated based solely on the individual’s characteristics while other times the household was the unit of analysis.

#### Social Capital

The Organisation for Economic Co-operation and Development (OECD) categorises measures of social capital into four groups; personal relationships, social network support, civic engagement and trust and cooperative norms [28]. They have identified over 1200 questions that are used as measures of social capital in surveys across the world. Specifically, they identified 138 questions that are felt to measure social capital in the European Social Survey, one of the national surveys included in the study. These types of question were found in data dictionaries of all the national surveys for which a data dictionary was available.

Overall, a measure of social capital was included in 12 data dictionaries reviewed. The most common form of social capital recorded was a person or their guardian’s marital status, which was noted in 10 data dictionaries. All had a different method of coding marital status. Examples of other measures of social capital included; personal relationships, who the subject lives with, care giving status (both whether they are cared for and if they are carers), questions about relationships with family and friends, computer ownership and neighbourhood environment.

### Other Stratifiers

All data dictionaries included age as a variable. Age groups were used for the reporting of findings for some data collections but all initially recorded age as a variable using either the date of birth of the individual or their age in years.

Nine data dictionaries recorded a measure of disability including three national data collections. All of these were registers of disability. All six data dictionaries from national surveys contained measures of disability. These contained measures of both physical and mental health including self-reported health.

Two data collections for which data dictionaries were available collected data on sexual orientation, both were national data collections. Some of the national surveys included questions about discrimination based on gender or sexual orientation but did not directly record sexual orientation or those who are not cisgender.

Other potential stratifiers that were identified in the data dictionaries reviewed but not further described included main language spoken, accommodation status, private health insurance and migrant status.

### Comments from Data Collection Managers

There was some correspondence of interest received from data collection managers in response to our requests for information. Several stated that they had never considered equity, while others had examined it in depth. One manager explained that they were concerned with equity, but that this was centred on ensuring that those with similar needs based on their diagnostic category have similar access to care rather than considering any social or demographic characteristics. Similarly, another manager felt that equity stratifiers might not be relevant for them because their programme was nationally implemented on a universal basis.

## Discussion

### Principal fFndings

This study found that while equity stratifiers were included in the routine collection of health and social care data, they were not routinely used to assess health inequalities. In addition, the definitions used for PROGRESS equity stratifiers were heterogeneous with some exceptions.

Stratifiers included were often not thought of in terms of their ability to detect inequity but instead for their role in service planning. Certain data collection managers did not feel that the consideration of equity was necessary to inform service planning. Such a blind spot for health equity measurement allows systematic inequalities to be reproduced rather than being detected and acted upon.

However, some data collections considered equity stratifiers in depth, including developing their own measures of inequality to best match their function. National surveys, as expected, collected information related to equity stratifiers more comprehensively and often had a developed theoretical basis for their uses.

### Strengths and Limitations

This study was intended to be an initial review of equity stratifiers used in health and social care data collections in Ireland. An analysis of the normative definitions of each individual stratifier would require significant theoretical consideration. It would also be dependent on the health topic and indicators being considered. Clearly some data collections in Ireland already define equity stratifiers for their own populations and at the level of the European Union draft legislation has been proposed to establish a common statistical framework across countries. However, a critique of the usefulness and validity of different stratifiers was outside of the scope of this study.

While no national or regional data collection contained all of the PROGRESS equity stratifiers, it is possible that some data collections do not need to concern themselves with stratifiers. Some data collections are disease registers for very rare diseases with only a handful of people diagnosed each year. Their small numbers may never have the power to able to detect inequalities and adding in additional equity stratifiers to their data collections may result in a poorer response rate without improving the output of the data collection.

This review looked mainly at individual level measures of equity but there may be important measures of equity which cannot be measured at the individual level. Many of these aspects of equity are considered in questions relate to social capital including a person’s neighbourhood environment and political outlook. They are mostly captured in the national surveys. Even with perfect information into an individual’s personal demographics, there may still be other factors which a health system needs to address in order to deliver an equitable service.

The data collections included in this analysis were those with a data dictionary available. It is unlikely that those collections that did not return a data dictionary or could not provide one to us would have a more comprehensive approach to monitoring inequality. The sample of data collections described in this study therefore likely overestimates the frequency of recording stratifiers for all health and social care data collections. As 28 data collections either had no data dictionary or a data dictionary was under review, there is an opportunity to recommend the inclusion of equity stratifiers in their data dictionaries for the future.

The main strength of this study was that it was able to use HIQA’s catalogue of health and social care data collections. While this list may not be complete, HIQA are uniquely placed as the statutory agency charged with setting the standards for the routine monitoring of health data in Ireland.

### The Future Role of Equity Stratifiers

Occupation was the stratifier which had the most homogenous definition between data collections and was mostly recorded in a Weberian manner [29]. This homogeneity is useful for comparing the effect of inequality across various health outcomes and over time. Agreed equity outcome measures also allow the effects of policy changes or interventions to be compared within and between populations.

This homogeneity could however be misleading if the underlying cause of inequality has not been correctly identified. For example, the European Social Survey also incorporates a Marxian class approach when recording occupation [27]. A Marxian approach incorporates class antagonisms produced by the social relationship between workers and capitalists. While this is a less commonly used method of recording occupation, there is an argument that can be made for and against both approaches yet the Weberian approach dominates.

This points to the importance of using the equity stratifiers which have a theoretical basis, are culturally appropriate and also which are acceptable for use by both patients and data collection managers. The definitions of the several equity stratifiers, including occupation, ethnicity and education, were commonly derived from the CSO. Using definitions decided upon by the CSO is of value as they are widely used, they allow for comparison and they undergo continuous revision including inviting public consultation. However, they are not chosen necessarily to reflect aspects of health inequality.

The findings of this study are not unique to data collections in Ireland, and there are hopefully possibilities for shared learning amongst those working in health inequalities and health information in other countries. The Canadian Institute of Health Information has developed a health inequalities toolkit. In order to do this they gathered groups of stakeholders together to prioritise potential stratifiers and then define them, balancing both practical and theoretical considerations before developing their equity toolkit [10]. It may be of use for other countries, including Ireland, to copy this process if there were a reasonable certitude that the agreed stratifiers would subsequently be widely adopted. This appears not to have been achieved so far in a Canadian context but changing data dictionaries of established data collections requires significant planning and agreement which takes time.

## Conclusions

There has been much discussion on tackling health inequalities in Ireland recently, however health and social care data collections do not always record the social groupings that are most commonly affected. Equity stratifiers are sometimes considered by the managers of health and social care data collections but this does not happen consistently. It is likely that by not considering equity stratifiers there may be health inequalities that are not measured and therefore cannot be acted upon.

While many causes of inequity are shared amongst countries and cultures, others act locally. Thus there is a need to advocate for agreed equity stratifiers for an Irish population, in order to measure and address health inequalities. Finally, while acknowledging the need for a more comprehensive approach to the measurement of inequalities, this will not be sufficient to tackle inequalities in years to come if the underlying systems that produce them go unchallenged.

## Data Availability

Data generated from this study are included in this published article or can be made available by emailing the corresponding author.

## Abbreviations

CSO: Central Statistics Office
DED: District electoral division
GMS: General Medical Services
HIQA: Health Information and Quality Authority
HIV: Human Immunodeficiency Virus
HSE: Health Service Executive
ISCO: International Standard Classification of Occupations
LGBTQ+: Lesbian, gay, bisexual, transgender, queer and others
WHO: World Health Organisation.
